# Morphological and Physiological Framework Underlying Plant Longevity in *Arabidopsis thaliana*

**DOI:** 10.3389/fpls.2020.600726

**Published:** 2020-11-05

**Authors:** Yukun Wang, Kie Kumaishi, Takamasa Suzuki, Yasunori Ichihashi, Nobutoshi Yamaguchi, Makoto Shirakawa, Toshiro Ito

**Affiliations:** ^1^Division of Biological Science, Graduate School of Science and Technology, Nara Institute of Science and Technology, Ikoma, Japan; ^2^RIKEN BioResource Research Center, Tsukuba, Japan; ^3^Department of Biological Chemistry, College of Bioscience and Biotechnology, Chubu University, Kasugai, Japan; ^4^Precursory Research for Embryonic Science and Technology, Japan Science and Technology Agency, Kawaguchi, Japan

**Keywords:** plant longevity, inflorescence meristem, stem cell, ROS, programmed cell death, *WUS*, *CLV3*, *Arabidopsis*

## Abstract

Monocarpic plants have a single reproductive phase, in which their longevity is developmentally programmed by molecular networks. In the reproductive phase of *Arabidopsis thaliana*, the inflorescence meristem (IM) maintains a central pool of stem cells and produces a limited number of flower primordia, which result in seed formation and the death of the whole plant. In this study, we observed morphological changes in the IM at cellular and intracellular resolutions until the end of the plant life cycle. We observed four biological events during the periods from 1 week after bolting (WAB) till the death of stem cells: (1) the gradual reduction in the size of the IM, (2) the dynamic vacuolation of IM cells, (3) the loss of the expression of the stem cell determinant *WUSCHEL* (*WUS*), and (4) the upregulation of the programmed cell death marker *BIFUNCTIONAL NUCLEASE1* (*BFN1*) in association with the death of stem cells. These results indicate that the stem cell population gradually decreases in IM during plant aging and eventually is fully terminated. We further show that the expression of *WUS* became undetectable in IM at 3 WAB prior to the loss of *CLAVATA3* (*CLV3*) expression at 5 WAB; *CLV3* is a negative regulator of *WUS*. Moreover, *clv3* plants showed delayed loss of *WUS* and lived 6 weeks longer compared with wild-type plants. These results indicated that the prolonged expression of *CLV3* at 4–5 WAB may be a safeguard that inhibits the reactivation of *WUS* and promotes plant death. Finally, through transcriptome analysis, we determined that reactive oxygen species (ROS) are involved in the control of plant longevity. Our work presents a morphological and physiological framework for the regulation of plant longevity in *Arabidopsis*.

## Introduction

How plants control their longevities is a conundrum that has puzzled botanists for decades (Thomas, 2013; Dijkwel and Lai, 2019). In the plant kingdom, the life spans of some species range from several weeks in annuals to thousands of years (Burian et al., 2016). Compared with those long-lived species, the monocarpic plant *Arabidopsis* [ecotype Landsberg *erecta* (L*er*)] has a relatively short life cycle, which is maintained at 50–70 days from seed germination to the formation of the next generation (Hensel et al., 1993). Although it is difficult to explain such large variations in longevity, an increasing number of studies have suggested that the variations are associated with the way that plants generate new cells (Heyman et al., 2014).

The shoot apex is a special part of the stem tissue that contains a limited number of stem cells, which is known as the crucial component of the shoot apical meristem (SAM) (Fletcher, 2002). The SAM has been characterized as a dynamic structure with self-renewing stem cells in the central zone (CZ) and organ primordia at the peripheral zone (PZ) (Steeves and Sussex, 1989). After the transition from vegetative growth to reproductive growth, SAM changes to an inflorescence meristem (IM). After seed germination, all further aboveground cells, tissues, and organs are regarded as descendants of stem cells in the CZ of the SAM and IM (Burian et al., 2016). Therefore, the SAM and IM are sometimes called the “fountain of youth” in plants (Baurle and Laux, 2003), and it is clear that stem cells hold the key to plant life span (Dijkwel and Lai, 2019); i.e., the activities of stem cells are a key factor in plant life span.

In recent decades, the formative and sustaining mechanisms of stem cells have been elucidated. It is well known that the stem cell population is maintained by the homeodomain transcriptional factor WUSCHEL (WUS), which is expressed in the organizing center (OC) and acts as the master regulator of stem cells. Subsequently, WUS moves from OC to the CZ through plasmodesmata and directly activates the expression of *CLAVATA3* (*CLV3*). Next, CLV3 peptides are secreted from cells and produce 13-amino-acid arabinosylated glycopeptides (CLV3 peptides) from the C-terminal region of CLV3. CLV3 peptides are diffused and bind the multiple extracellular receptor-like kinases including CLAVATA1 (CLV1), resulting in the repression of the expression of *WUS* and the restriction of the expression region of *WUS*. These molecular components form the WUS–CLV negative feedback loop to control the dynamic status of stem cells (Brand et al., 2000; Schoof et al., 2000). Although it is well known how plants control stem cell formation and maintenance, less is known regarding stem cell fate in plant aging. Recently, a study presented novel findings at the genetic level suggesting that the MAD-box gene *FRUITFULL* (*FUL*) directly and negatively regulates *APETALA2* (*AP2*) expression in the IM and maintains the temporal expression of *WUS*, thereby regulating stem cell maintenance and controlling plant life span in *Arabidopsis* (Balanzà et al., 2018). Very recently, it was reported that auxin export from proximal fruits drives arrest in competent inflorescences and that the arrest of IMs is local and uncoordinated between different branches (Ware et al., 2020). However, to obtain a better understanding of the aging-dependent stem cell fate in *Arabidopsis*, more evidence at the morphological, physiological, and molecular levels is necessary.

In recent years, the roles of programmed cell death (PCD) in regulating plant growth and development have been extensively studied (Daneva et al., 2016). As one type of PCD, developmental PCD (dPCD) has become a heavily researched topic, and its roles are largely revealed in the vegetative and reproductive stages in plants (Daneva et al., 2016). To date, the dPCD process has shown functions in the cell death of nucellar tissue (Radchuk et al., 2006, 2011; Yin and Xue, 2012), antipodal cell death (Groß-Hardt et al., 2007; Moll et al., 2008), tapetum cell death (Sorensen et al., 2003; Phan et al., 2011), xylogenesis (Yamaguchi et al., 2010), lateral root cap differentiation (Fendrych et al., 2014; Olvera-Carrillo et al., 2015), and organ abscission and dehiscence (Lers et al., 2006; Farage-Barhom et al., 2008; Kasaras and Kunze, 2010; Bar-Dror et al., 2011). During the dPCD process in different types of plant organs, several dPCD-associated genes that commit cells to PCD, including *BIFUNCTIONAL NUCLEASE1* (*BFN1*) and *PLANT ASPARTIC PROTEASEA3* (*PASPA3*), are expressed (Fendrych et al., 2014; Olvera-Carrillo et al., 2015). In particular, *BFN1* is activated in almost all senescent or dead tissues and thus is used as a critical marker gene to monitor the dPCD process (Perez-Amador et al., 2000; Farage-Barhom et al., 2008). The PCD process can be triggered via multiple signaling pathways, and reactive oxygen species (ROS) are among the key components of this process. Many studies have investigated whether ROS homeostasis is correlated with the regulation of cell death in plants, and abnormal ROS accumulation can trigger the PCD process (Hu et al., 2011; Luo et al., 2013; Zheng et al., 2019). However, it has not been determined whether the ROS-mediated PCD process occurs during stem cell life span. In the present study, we observed morphological changes of the IM at cellular and intracellular resolutions until the end of the plant life cycle.

We observed that the gradual reduction of IM size and the dynamic vacuolation of IM cells began at 1 week after bolting (WAB). Moreover, the expression of *WUS* was dynamically reduced until 3 WAB, and the upregulation of the PCD marker *BFN1* was detected at 5 WAB and was associated with death of stem cells. These results indicate that the stem cell population in the IM is decreased during plant aging. In addition, RNA sequencing (RNA-seq) and imaging analyses revealed that the ROS module was involved in the death of IM cells. Finally, we proposed that the aging of the IM in *Arabidopsis* consists of three phases. The results of this study may help to elucidate the regulatory mechanism governing plant longevity in *Arabidopsis*.

## Materials and Methods

### Plant Materials and Growth Conditions

All *Arabidopsis thaliana* seed stocks used in this study were in the L*er* background. The *clv3-2* mutant was described previously (Clark et al., 1995). The reporter lines *proWUS*:*GFP-ER*, *proCLV3*:*GFP-ER*, and *proWUS*:*GUS* were reported previously (Lenhard and Laux, 2003; Gordon et al., 2007; Rodriguez et al., 2016; Sun et al., 2019). *Arabidopsis* seeds were sown in pots containing vermiculite and Metro-Mix and incubated at 4°C in the dark for 3 days to promote germination. All plants were cultured in an illumination incubator (Biotron, LPH-411SP, Japan) under a 16-h light (100 μmol m^–2^ s^–1^)/8-h dark light cycle with 60% humidity and at 22°C.

### Phenotypic Definitions and Measurements

To observe the development of each *Arabidopsis* plant precisely during aging, we applied WAB as the temporal unit (Balanzà et al., 2018). When the stem length approached 1 cm, this time point was defined as the initiation of bolting (Noodén and Penney, 2001). For the counting of flower numbers on the primary stem, the siliques and flowers older than stage 7 were counted. The flower stage was referenced to the criterion described by Smyth et al. (1990). The measurement of IM size was estimated by measuring the IM circumference from a maximum diameter (Daum et al., 2014). The IM circumference was defined by the boundary between IM and the floral primordium. The cells with a large vacuole in the IM domain were judged by the area ratio between the vacuole and the whole cell. If the area ratio was over 40% in a cell (using FIJI to measure the size of the cell and vacuole), then the cell was considered to be a cell with a large vacuole. To ensure that the observed cells were stem cells, a total of 18 and 6 cells in the stem cell layers were observed in the wild type (WT) and *clv3-2* mutant, respectively. In *clv3-2* mutant, we select cells in L1 because layers without L1 were disorganized. The ratio of cells with large vacuoles was the ratio between the number of cells with large vacuoles and total cells observed. The measurements of IM circumference and diameter were performed using FIJI (v1.50b)^1^ (Schindelin et al., 2012). The morphological observations of inflorescences on primary shoots were performed using an optical camera (Canon EOS 600D).

### Scanning Electron Microscope

Inflorescences of primary WT shoots were fixed in formalin–acetic acid–alcohol (FAA) solution overnight at room temperature and dehydrated with an ethanol and acetone series. Critical point drying with liquid CO_2_ and a gold coating were performed using EM CPD300 (Leica, Germany) and E-1010 (Hitachi, Japan), respectively. The inflorescences were observed using an S-4700 scanning electron microscope (SEM) (Hitachi, Japan) with an accelerating voltage of 15 kV.

### Transmission Electron Microscope

For transmission electron microscopy (TEM) observation, inflorescences of primary shoots of *Arabidopsis* WT plants and *clv3-2* mutant plants were harvested at each time point (1–6 WAB). The methods of sample fixation and sectioning were described previously (Yamaguchi et al., 2018). Photographs were taken using an H-7100 TEM (Hitachi, Japan).

### GUS Staining and Tissue Sectioning

Inflorescences of primary shoots of reporter lines were fixed in 90% acetone for 15 min at room temperature, rinsed with double-distilled water, and subsequently stained with GUS staining solution. The staining method was described previously (Shirakawa et al., 2014). Tissue sectioning was performed as described previously (Yamaguchi et al., 2018). The slides were stained with 0.05% neutral red (Wako Chemicals, Japan) or 0.01% toluidine blue (Wako Chemicals, Japan).

### Confocal Microscopy

To observe the green fluorescent protein (GFP) signal in the longitudinal sections of the IMs on *proWUS*:*GFP-ER* primary shoots, the floral buds older than stage 7 were removed with tweezers under a light microscope, and then the IMs were embedded into 5% agar (Difco) and sliced with a Liner Slicer PRO7 vibratome (Dosaka, Japan) (Yamaguchi et al., 2018). The resulting tissue sections were immersed in moderate volumes of 1/10 Murashige and Skoog (MS) solution on glass slides. The GFP signal was immediately observed under an FV 1000 (Leica, Germany) microscope with FV10-ASW software. To detect the GFP signal in *proCLV3*:*GFP-ER*, the IMs on primary shoots were immersed in moderate volumes of 1/10 MS containing FM4-64 (Thermo Fisher, 5 μg/ml) on glass slides and covered with coverslips for 10 min. The images of the transverse orientation (*XY* axis) were taken with an inverted ZEISS LSM710 confocal laser scanning microscope. The images of longitudinal orientation were reconstructed from Z-stack images along the *XY* axis by ZEN software. GFP was excited with the 488-nm argon laser, and the emission was detected between 495 and 545 nm. FM4-64 was excited with the 561-nm laser, and the emission was detected between 570 and 620 nm (Shi et al., 2018).

### RNA-seq

The IMs (including floral buds up to stage 7) on primary shoots of WT at 2 and 4 WAB were collected as RNA-seq samples. For each sample, at least 50 individual IMs were collected under microscopes using sterile forceps and frozen in liquid nitrogen immediately. The RNeasy Plant Mini Kit (QIAGEN, Germany) was used to extract total RNA from the four biological replicates. DNA was removed using the RNase-Free DNase Kit (QIAGEN, Germany). The methods of library construction and sequencing were described previously (Townsley et al., 2015; Uemura et al., 2018). Briefly, the mRNA was fragmented using magnesium ions at elevated temperatures, after which the polyA tails of mRNA were primed using an adapter-containing oligonucleotide for cDNA synthesis with DNA Polymerase I (Thermo Fisher Scientific). The 5′ adapter addition was performed using breath capture to generate strand-specific libraries. The final PCR enrichment was performed using oligonucleotides containing the full adapter sequence with different indexes and Phusion High-Fidelity DNA Polymerase (New England Biolabs). The cleanup and size selection of the resulting cDNA were performed using AMPure XP beads (Beckman Coulter). The size distribution and concentration of the libraries were measured using agarose gel electrophoresis and a microplate photometer, respectively, to enable the pooling of libraries for Illumina sequencing systems. The libraries were sequenced by NextSeq 500 (Illumina). The produced bcl files were converted to fastq files by bcl2fastq (Illumina). Mapping to the *A. thaliana* reference (TAIR10) was conducted using Bowtie with the following options “−−all −−best −−strata −−trim5 8.” The number of reads mapped to each reference was counted. After normalization, the false discovery rate (FDR) and fold change were calculated using the edgeR package for R (Wu et al., 2019). The differentially expressed genes (DEGs) were isolated with a log_2_ fold change ≥1 or log_2_ fold change ≤−1 and FDR < 0.05 (Wang et al., 2018). Gene Ontology (GO) term enrichment analysis of DEGs was carried out using Blast2GO (*q*-value ≤ 0.05). Kyoto Encyclopedia of Genes and Genomes (KEGG) pathway analysis was performed (*q*-value ≤ 0.05) using BlastX searches against the KEGG pathway database (Wang et al., 2018).

### Reverse-Transcription PCR and Quantitative RT-PCR

The RNeasy Plant Mini Kit (QIAGEN, Germany) was used to extract total RNA. The RNase-Free DNase Set (QIAGEN, Germany) was used to eliminate the contamination of genomic DNA in RNA samples. Reverse-transcription PCR was performed using PrimeScript^TM^ RT Master Mix (Takara, Japan). Quantitative RT-PCR was applied as described previously (Yamaguchi et al., 2018). Arabidopsis *ACTIN2* (*AT3G18780*) was used as the internal reference. Each experiment was repeated three times with four technical replicates. The relative expression level of each gene was calculated using the 2^–ΔΔ*Ct*^ method (Livak and Schmittgen, 2001).

### Plasmid Construction and Plant Transformation

To generate the *proBFN1*:*GUS-GFP* construct, a genomic DNA fragment covering a sequence 2.0 kb upstream of the *BFN1* translation start site was subcloned into the pENTR/D-TOPO vector according to the manufacturer’s protocol (Thermo Fisher, Germany). After confirmation by sequencing, the plasmid containing the fragment was employed in the LR reaction with the pBGWFS7 vector, which was a gateway vector containing GUS and GFP coding sequences, according to the manufacturer’s protocol (Gateway^TM^ LR Clonase^TM^ II Enzyme Mix, Thermo Fisher, Germany). The recombinant construct *proBFN1*:*GUS-GFP* was transformed into *Agrobacterium tumefaciens* strain GV3101 by using the freeze–thaw method. The *Agrobacterium*-mediated floral dip method was performed to perform transgene analysis (Zhang et al., 2006). T1 seeds were collected and screened using the chemical Basta. More than 20 T1 plants were obtained, and the representative line was chosen for further study.

### DAB Staining

The method of 3,3′-diaminobenzidine (DAB) staining of IM was described previously (Zeng et al., 2017). The chlorophyll in stained IM tissues was discolored in boiling ethanol (ethanol:glycerin:glacial acetic acid = 3:3:1).

### Fluorescein Diacetate and Propidium Iodide Staining

Fluorescein diacetate (FDA) (Sigma) was dissolved in acetone to produce a 1 mg/ml stock solution. The working solution (20 μg/ml) of FDA was prepared by diluting 20 μl of the stock solution in 1 ml of 1/10 MS solution. By dissolving 1 mg propidium iodide (PI) in 1 ml sterile water, 1 mg/ml stock solution of PI (Sigma) was prepared. By diluting 10 μl of the stock solution in 1 ml of 1/10 MS solution, 10 μg/ml working solution of PI was prepared. IMs without any dissection were stained for 20 min. Then, samples were put on glass slides and covered with coverslips. FDA was excited with the 488-nm laser line of the argon laser, and the emission was detected between 495 and 545 nm, and PI was excited with a 561-nm diode laser and detected between 580 and 680 nm (Gao et al., 2018). The images of the transverse orientation (*XY* axis) were taken with an inverted ZEISS LSM710 confocal laser scanning microscope. The images of longitudinal orientation were reconstructed from the Z-stack images along the *XY* axis by the ZEN software.

### Data Statistics and Availability

In this study, one-way ANOVA followed by the Tukey–Kramer test (*p* < 0.01) or Student’s *t*-test (two-tailed, *p* < 0.05) was performed to detect differences as required. All primers used in this study are listed in Supplementary Table 1. The RNA-seq data sets were submitted to the DNA Data Bank of Japan with the accession number DRA010789.

## Results

### Growth and Termination of the Primary Inflorescence

**FIGURE 1:**
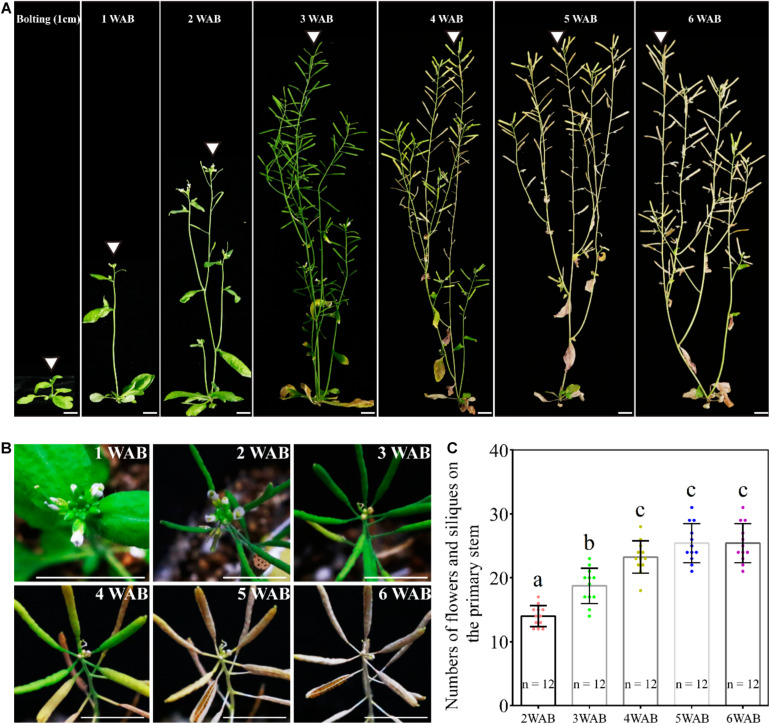
Growth and termination of the primary inflorescence of *Arabidopsis thaliana*, Landsberg *erecta*. **(A)** Photographs of plant morphologies at the bolting time point (the stem length was reached at 1 cm) and from 1 to 6 WAB. White triangles mark the primary inflorescence at each time point. Scale bars = 1 cm. **(B)** Serial top views of the primary inflorescences at 1–6 WAB. Twelve individual plants were observed, and representative images are shown. Scale bars = 1 cm. **(C)** The quantification of numbers of flowers and siliques on primary stems of 12 individual plants. The flowers beyond stage 7 were counted. Dots represent numbers of flowers and siliques from each sample. Error bars represent SD. One-way ANOVA followed by the Tukey–Kramer test was performed (*p* < 0.01). Different letters indicate significant differences, while the same letters indicate non-significant differences.

In our growth conditions, WT L*er* plants were bolted 35 days after germination, and they reached the maximum height at 3 WAB (Figure 1A). After 3 WAB, multiple siliques were produced, and aging began with the change in plant color from green to brown (Figures 1A,B). At 4 WAB, aging of plants progressed, resulting in some siliques starting to turn yellow. At 5 and 6 WAB, mature siliques were opened, and plants dispersed seeds (Figure 1B). During the whole life cycle of plants, the sum of the number of both flowers and siliques increased continuously until 4 WAB (Figure 1C; *p* < 0.01, Tukey–Kramer test).

### Gradual Decrease in the Size of the IM

**FIGURE 2:**
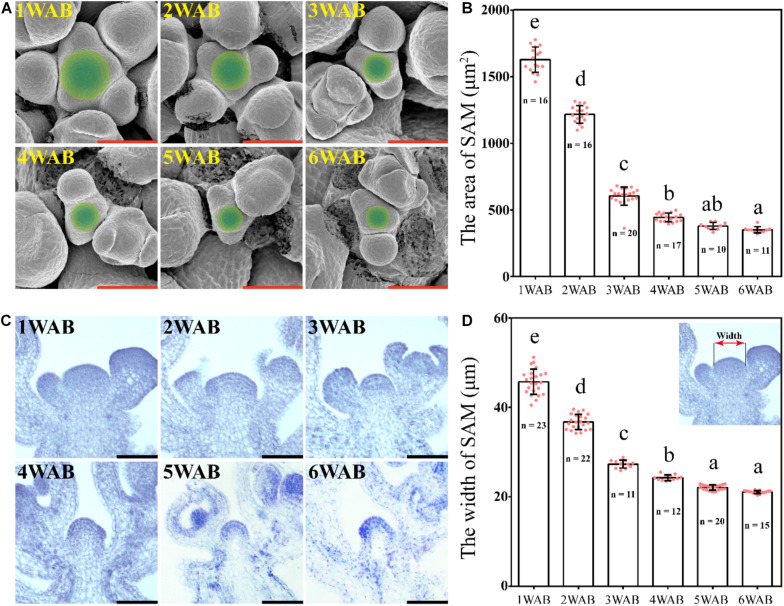
Size of the inflorescent meristem gradually decreased. **(A)** The top views of IM domains of wild type from 1 to 6 WAB. SEM was used. Green circles indicate IM domains (including CZs and PZs) (Daum et al., 2014). Scale bars = 40 μm. **(B)** The area of IM domains of wild type from 1 to 6 WAB. The number (n) of observed samples at each time point is shown. Dots represent the area of IM from each sample. Error bars indicate SD. One-way ANOVA followed by the Tukey–Kramer test was performed (*p* < 0.01). Different letters indicate significant differences, while the same letters indicate non-significant differences. **(C)** Longitudinal views of IMs of wild type from 1 to 6 WAB by using histologic sections. Scale bars = 40 μm. **(D)** The width of IM domains of wild type from 1 to 6 WAB. The image at the top right corner indicates the definition of the SAM width (Daum et al., 2014). The number (n) of observed samples at each time point is shown. Dots represent the width of IM from each sample. Error bars indicate SD. One-way ANOVA followed by the Tukey–Kramer test was performed (*p* < 0.01). Different letters indicate significant differences, while the same letters indicate non-significant differences.

We hypothesized that IM activity was limited and lost at the end of the plant life cycle because the total number of flowers and siliques was controlled (Figure 1). To examine whether IM size is reduced during aging, we measured IM sizes by using SEM from 1 to 6 WAB (Daum et al., 2014; Figures 2A,B). IM sizes were notably reduced from 1 until 6 WAB, and IM sizes at 5 and 6 WAB were minimal (Figure 2B; *p* < 0.01, Tukey–Kramer test). Consistent with these results, the maximum width of IM in cross-sections was also notably reduced until 5 WAB, and the IM width at 5 and 6 WAB was minimal (Figures 2C,D; *p* < 0.01, Tukey–Kramer test). At 4 WAB, the total number of flowers and siliques reached a maximum number (Figure 1C). Taken together, these results suggested that IM activity before 4 WAB is required for the production of seeds. These results suggested that the IM gradually shrinks during the aging of plants.

### Dynamic Transition of Intracellular Structures of Stem Cells in L1 and L2 of IM

**FIGURE 3:**
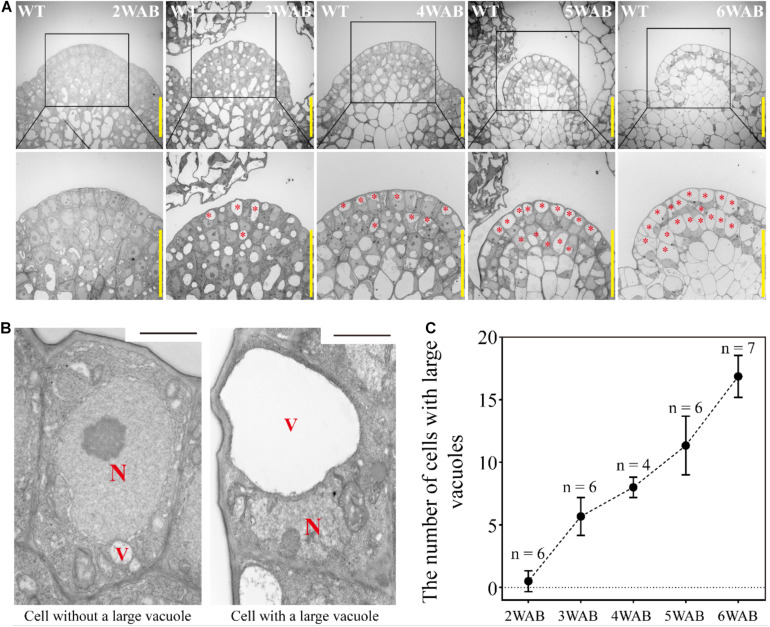
Dynamic transition of intracellular structures of stem cells in L1 and L2 of IM. **(A)** The intracellular ultrastructures of stem cells in L1 and L2 of IM of wild type from 2 to 6 WAB by using TEM. Images in the **lower panels** indicate the magnified images of the black-box area in the **upper panels**. Red stars indicate cells with large vacuoles that occupied over 40% of the cell size. Scale bars = 20 μm. **(B)** Representative images of cells without or with a large vacuole in IM. N, nucleus; V, vacuole. Scale bars = 1.5 μm. **(C)** The number of cells with large vacuoles in IM of wild type from 2 to 6 WAB. The number of samples at each time point is shown. Error bars indicate SD.

At 4 WAB, the IM size was almost minimal (Figure 2). Next, we analyzed the transition of the ultrastructure of cells in both L1 and L2 (L1/2) of IM by using TEM. At 2 WAB, all cells in L1/2 were filled with electron-dense materials (dark gray color), and intracellular spaces were occupied primarily by a large nucleus and cytoplasm (Figures 3A,B left). Combined with the expression data of stem cell markers (described below), these cells have high proliferative potential. At 3 WAB, some of the cells in L1/2 had large vacuoles whose sizes were nearly equal to the sizes of the nuclei (Figures 3A,B right). After 3 WAB, the numbers of cells with large vacuoles increased until 6 WAB (Figures 3A,C). Almost all cells in the IM had a central large vacuole at 6 WAB. Large vacuoles are one of the indicators of differentiated cells. Combined with the results regarding plant growth (Figure 1) and IM size (Figure 2), these results suggested that even stem cells in the IM were getting differentiated and IMs lost their proliferative activity at approximately 4 WAB.

### Expression Patterns of Stem Cell Markers in the IM Domain During Aging

**FIGURE 4:**
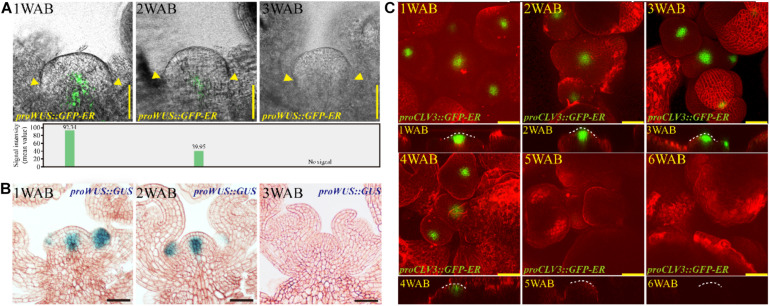
Expression patterns of stem cell markers in the IM domain during aging. **(A,B)** The spatiotemporal expression patterns of *WUS* from 1 to 3 WAB. The *proWUS*:*GFP-ER* and *proWUS*:*GUS* lines were used in **(A,B)**, respectively. The GFP signal intensity was quantified and is shown in the **lower panel** of **(A)**, and yellow triangles denoted boundaries of SAMs. Scale bars = 25 μm in **(A,B)**. **(C)** The spatiotemporal expression patterns of *CLV3*. The *proCLV3*:*GFP-ER* line was used. The top view of confocal images is shown in the **upper panel**, and the side view is shown in the **lower panel**. To visualize the outline of cells, we stained IM with FM4-64 dye (red). White dotted lines indicate SAM shapes. Scale bars = 25 μm.

How do plants lose the proliferative activity of IM at approximately 4 WAB? To examine how the morphological changes and stem cell marker gene expression levels during aging were coordinated, we examined the dynamic expression patterns of two stem cell marker genes, *WUS* and *CLV3*, during the aging of the IM (Figure 4 and Supplementary Figure 5A). *CLV3* was expressed at cells in L1/2 of the CZ of the IM, and *WUS* was expressed at the OC, which is located below the CZ (Brand et al., 2000; Schoof et al., 2000; Figure 4). The expression level of *WUS* at 1 WAB was highest, the expression of this gene gradually decreased during aging until 3 WAB, and no expression was detected at 3 WAB in either the GFP reporter or the GUS reporter (Figures 4A,B). After 3 WAB, *WUS* expression was not recovered (Supplementary Figure 1). Combined with the morphological data, these results suggested that IM cells began to lose stem cell/proliferative activity after 1 WAB because they began to reduce their expression of *WUS*. This hypothesis is consistent with the results demonstrating that the IM size at 3 WAB was less than 50% of the IM size at 1 WAB (Figure 2B) and that some IM cells at 3 WAB had a large vacuole (Figure 3). Compared with *WUS*, the expression of *CLV3* was maintained longer than 4 WAB, and no expression was detected at 5 WAB in the GFP reporters (Figure 4C). These results suggested that the expression period of *CLV3* was 2 weeks longer than that of *WUS*. A similar observation was reported by using *proCLV3*:*GUS* lines (Balanzà et al., 2018). These results suggested that *CLV3* might play an additional role in the regulation of plant longevity (described below).

### ROS Are Involved in the Death of Stem Cells in the IM

**FIGURE 5:**
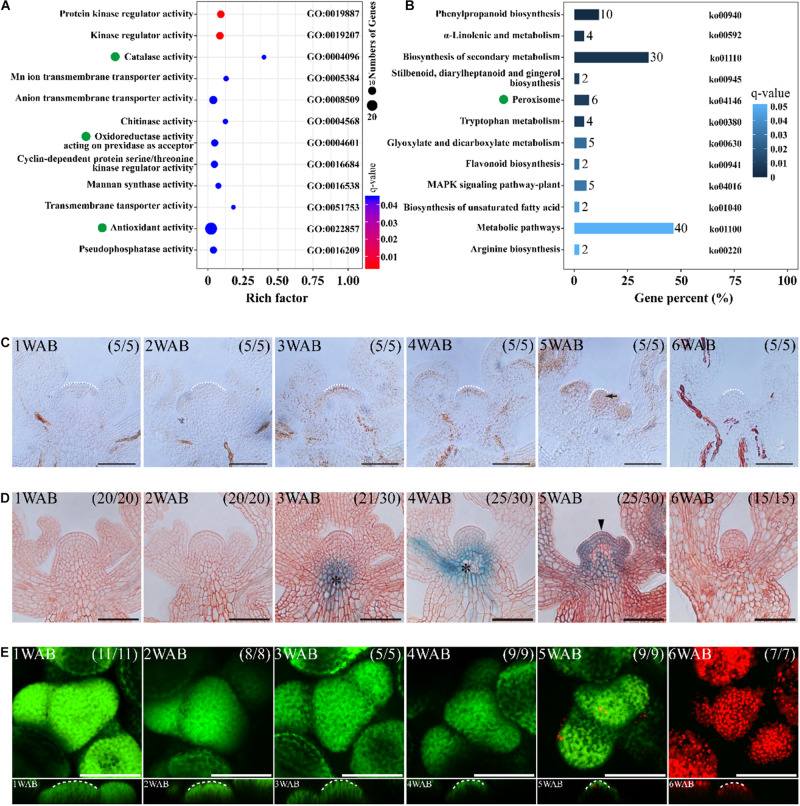
ROS are involved in the death of stem cells in IM. **(A,B)** Omics analyses of DEGs of IMs between 2 and 4 WAB. RNA-seq was performed with four biological replicates of each sample. In both analyses, ROS-related genes were enriched. **(A)** GO term enrichment of the DEGs of IMs between 2 and 4 WAB. Green dots indicate ROS-related GO terms (catalase activity, oxidoreductase activity, and antioxidant activity). **(B)** KEGG pathway analysis of the DEGs. Green dots denote ROS-related pathways (peroxisome). **(C)** DAB staining of IM from 1 to 6 WAB. White dotted lines indicate IM shape. The black arrow indicates the accumulation of the H_2_O_2_ signal in IM. Scale bars = 50 μm. **(D)** The spatial–temporal expression patterns of a PCD marker gene, *BFN1*, in IM from 1 to 6 WAB. Scale bars = 50 μm. The black arrowhead indicates the GUS signal in stem cells in the CZs and peripheral cells. Black asterisks indicate GUS outside IM (vascular tissues). **(E)** FDA/PI staining of IM from 1 to 6 WAB. The top view of confocal images of FDA (green) and PI (red) signals is shown in the **upper panel**. The side view is shown in the **lower panel**. FDA-stained cells (in green) are alive, and PI-stained cells (in red) are dead. White dotted lines indicate SAM shapes. Scale bars = 50 μm.

We hypothesized that the dynamic changes of gene expressions might occur between 2 and 4 WAB because the size of IMs was reduced dynamically, the intracellular vacuolations were progressed, and the expression of *WUS* was lost during these 2 weeks. To clarify the transcriptional dynamics of plant aging, we compared RNA-seq profiles of four independent IM samples between 2 and 4 WAB. We isolated 547 DEGs, including 492 upregulated DEGs and 55 downregulated DEGs (Supplementary Figure 2). To understand the putative functions of these DEGs, we performed GO term enrichment and KEGG pathway analyses. Notably, we found that some of the DEGs were specifically clustered into ROS-related GO terms and KEGG pathways. Five DEGs were clustered into “catalase activity” (GO: 0004096), seven DEGs were gathered into “oxidoreductase activity acting on peroxide as acceptor” (GO: 0004601), and 25 DEGs were enriched into “antioxidant activity” (GO: 0022857) (Figure 5A). Based on the results of KEGG pathway analysis, we found six DEGs involved in the KEGG pathway “peroxisome” (ko04146) (Figure 5B). By removing the redundant DEGs, we obtained eight ROS-related DEGs (Table 1). By qPCR analysis, we confirmed that all eight DEGs were significantly upregulated at 4 WAB compared with those at 2 WAB (Supplementary Figure 3). These results were shown to be highly consistently with the RNA-seq data (Table 1) in which ROS-related genes are upregulated during aging of IMs. In addition, in keeping with the results of the *proCLV3*:*GFP-ER* reporter lines, our qPCR analysis indicated a significant reduction in the expression levels of *CLV3* (Supplementary Figure 3).

**TABLE 1:** ROS-related DEGs.

**Gene ID**	**Gene Name**	**Log_2_FC**	**Expression pattern**	**Adjusted *p*-value**	**GO term or KEGG pathway**	**Annotation**
AT1G20620	CAT3	1.10	Up	4.82E–02	GO:0004096	catalase 3
AT1G20630	CAT1	1.25	Up	3.14E–02	GO:0004096	catalase 1
AT1G73680	DOX2	1.39	Up	2.65E–02	GO:0004601	alpha dioxygenase
AT4G11600	GPX6	1.32	Up	1.58E–02	GO:0004601	glutathione peroxidase 6
AT4G16760	ACX1	1.45	Up	6.45E–03	GO:0022857	acyl-CoA oxidase 1
AT4G37530	PRX51	3.99	Up	1.87E–02	ko04146	peroxidase superfamily protein
AT5G06720	PRX53	7.16	Up	3.53E–02	ko04146	peroxidase 2
AT5G64110	PRX70	1.89	Up	2.41E–02	ko04146	peroxidase superfamily protein

It has been reported that ROS regulates PCD in both plants and animals. Therefore, we hypothesized that ROS-mediated PCD occurred after IM maturation at 4 WAB. To elucidate the spatiotemporal accumulation pattern of the ROS hydrogen peroxide (H_2_O_2_), we performed DAB staining in cross-sections of IM from 1 to 6 WAB. We observed pronounced accumulation of hydrogen peroxide in the IM region at 5 WAB when IM cells were fully matured with a large vacuole (Figure 5C). Next, we examined the expression of a PCD marker gene, *BFN1*, which mediates the degradation of nucleic acids (Figure 5D). In the stem cells, we found a notable expression peak of BFN1 at 5 WAB, while vasculature expression started earlier at 3 WAB (Figure 5D). These results suggested that ROS and *BFN1* were involved in PCD of the stem cells. Next, we observed cell death in the IM region by FDA/PI staining. At 5 WAB, some cells were dead and thus were stained by PI in the IM region; however, the majority of cells were alive (Figure 5E and Supplementary Figure 5B). In contrast, at 6 WAB, all cells were dead (Figure 5E and Supplementary Figure 5B). These results suggested that PCD in IM cells was initiated at 5 WAB and was completed before 6 WAB.

### *clv3* Exhibited a Longer Longevity Phenotype Than the WT

**FIGURE 6:**
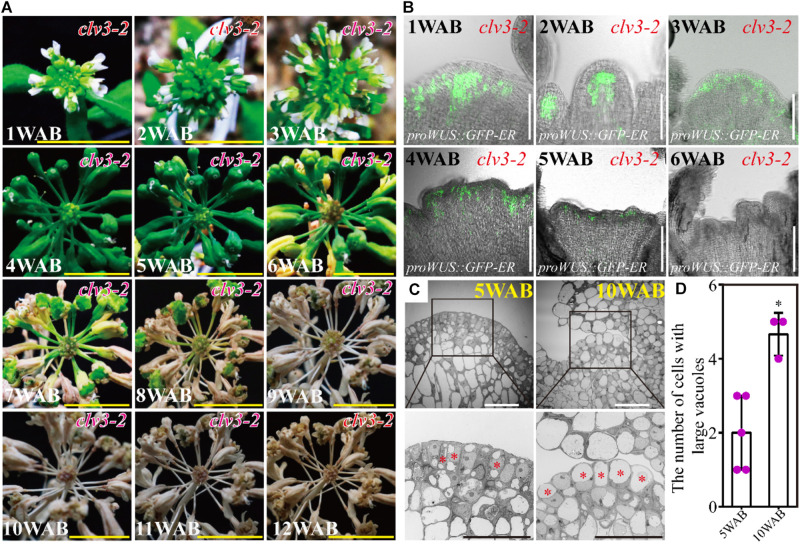
*clv3-2* mutant showed a prolonged life span of IM. **(A)** Morphological changes in *clv3-2* inflorescences from 1 to 12 WAB. Scale bars = 1 cm. **(B)** Spatial–temporal expression patterns of *WUS* in IM of *clv3-2*. The *proWUS*:*GFP-ER* reporter line was used. Scale bars = 100 μm. **(C)** The intracellular ultrastructures of stem cells in L1 and L2 of IM of *clv3-2* at 5 and 10 WAB by using TEM. Images in the **lower panels** indicate the magnified images of the black-box area in the **upper panels**. Red stars indicate cells with large vacuoles that occupied over 40% of the cell size. Scale bars = 20 μm. **(D)** The number of cells with large vacuoles in *clv3-2* IM at 5 and 10 WAB. Dots represent the vacuolate cell numbers at each time point. Error bars denote SD. Two-tailed Student’s *t*-test was performed. ^∗^*p* < 0.05.

The *clv3* mutants produce increased number of flowers (Clark et al., 1995), and thus, we examined the plant morphology and the longevity of *clv3-2* mutants until 12 WAB. As previously reported, *clv3-2* mutants exhibited enlarged meristematic tissues, resulting in an increased number of flowers and fruits (Figure 6A). In addition to these phenotypes, *clv3-2* mutants lived 6 weeks longer than WT plants after bolting (Figure 6A; please compare with Figure 1B). At 4 WAB, when some siliques started to turn yellow in WT plants, all siliques of *clv3-2* mutants kept a green color. At 5 WAB, when some mature siliques were opened in WT plants, some siliques of *clv3-2* mutants started to turn yellow. At 6 WAB, when whole wild-type plants are dead, *clv3-2* mutants are alive and exhibit green-colored IM. From 7 to 11 WAB, the brown region was expanded in *clv3-2* mutants. At 12 WAB, whole *clv3-2* mutant plants were dead. Consistent with this finding, the expression window of *WUS* was also 3 weeks longer than that of the WT (Figure 6B; please compare with Figure 4A; WT plants expressed *WUS* until 2 WAB, and *clv3-2* mutant plants expressed *WU*S until 5 WAB). In addition, *clv3* mutants exhibited an increase in differentiated IM cells with large central vacuoles until 10 WAB compared with those observed at 5 WAB (Figures 6C,D and Supplementary Figure 4). These results suggested that *clv3* mutants possessed a longer longevity phenotype. These results suggested that *CLV3* is a safeguard that inhibits the longer expression window of *WUS* at 3–5 WAB by shutting down *WUS* expression at the correct time.

## Discussion

### Phase Transition of Stem Cells of IM During Aging in *A. thaliana*

**FIGURE 7:**
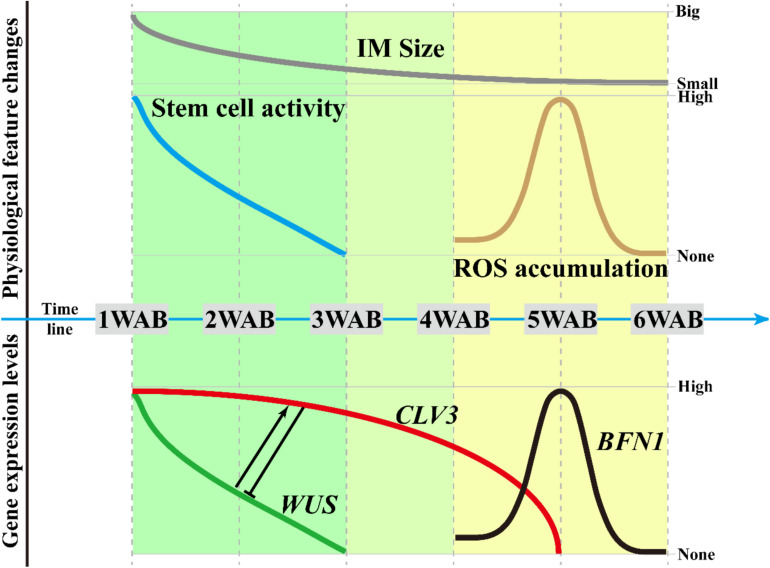
Regulatory framework of the IM life span. Based on this study, we defined three phases of the IM life span. In the first phase (from 1 to 3 WAB), along with the reduction in *WUS* expression in the CZ of the IM, the stem cell activity and size of the IM domain gradually decreased. At 3 WAB, *WUS* promoter activity is fully terminated. Next, from 3 to 4 WAB (second phase), the transition of the intracellular ultrastructure of IM cells progresses, resulting in an increase in the number of cells with a large vacuole. These cells may be ready to be killed by PCD. At the same time, however, the expression level of *CLV3* is still maintained because *CLV3* may have a role in inhibiting the reactivation of *WUS*. In the third phase (from 4 to 6 WAB), ROS accumulation and the expression of the programmed cell death indicator *BFN1* were observed in IM at 5 WAB. They may promote the death of cells with a large vacuole in IM, and almost all cells in IM are stained by PI at 6 WAB.

In this study, by using the *Arabidopsis* L*er* accession, we determined the morphological changes (Figures 1, 2), intracellular ultrastructures (Figure 3), and changes in gene expression (Figures 4, 5) of the IM during aging. From these results, we proposed three different phases in the aging of *Arabidopsis*, which are summarized in Figure 7. In the first phase (green in Figure 7; 1–3 WAB), along with the reduction in *WUS* expression in the CZ of the IM (green line), the stem cell activity (blue line) and size of the IM domain (gray line) gradually decrease. At 3 WAB, the *WUS* promoter activity is fully terminated at the IM domain.

In the second phase (light green in Figure 7; 3–4 WAB), the transition of the intracellular ultrastructure of IM cells progresses continuously, resulting in an increase in the number of cells with large vacuoles. These cells may be ready to be killed by PCD. At the same time, the expression level of *CLV3* (red line) remains high. Since *CLV3* has a role in inhibiting *WUS* expression, CLV3 may function as a component of the putative safeguard system to prevent reactivation of *WUS*. Consistently, *clv3* mutants exhibited 3 weeks longer expression window of *WUS* and lived 6 weeks longer than WT plants after bolting. Consistent with this, it was reported that the leaf longevity in *clv3-2* plants was 20–30 days longer than that in WT plants in combined short-day and long-day culture conditions (Noodén and Penney, 2001).

It would be interesting to determine which factor(s) promote *CLV3* expression after the loss of *WUS* at 3-WAB expression because WUS is a known critical activator for *CLV3*. Unknown transcriptional factor(s) may maintain the expression of *CLV3* after 3 WAB. However, we could not exclude the possibility that WUS proteins still exist until 4 WAB and promote the expression of *CLV3* directly. To investigate this possibility, confocal microscopy with ultrahigh sensitivity is needed because the WUS protein is unstable.

At the third phase (yellow in Figure 7; 4–6 WAB), ROS accumulation (brown line) and the expression of the PCD indicator *BFN1* (black line) were observed in IM at the middle of phase 3, that is, 5 WAB. ROS accumulation and *BFN1* expression may promote the death of cells with large vacuoles in the IM because almost all cells in the IM are stained by PI at 6 WAB. These phases may be useful to future research attempting to identify mutants with defects in the progression of aging.

### In Arabidopsis, the Final Fate of Stem Cells in the IM May Be PCD

It is well known that senescent cells often exhibit large vacuoles (Rhinn et al., 2019), that cell vacuolization means terminal differentiation, and that such cells have lost their proliferative and differentiated abilities (Shubin et al., 2016). Moreover, the vacuole is an executor of PCD (Hara-Nishimura and Hatsugai, 2011). In this study, we found that the number of stem cells with large vacuoles increased during the life span (Figure 3). In addition, we detected the expression peak of a PCD marker gene, *BFN1*, at 5 WAB (Figure 5D), and we observed PI signals in stem cells in layers 1 and 2 at 6 WAB (Figure 5E). These results indicate that the final fate of stem cells is age-induced dPCD. In plants, age-induced dPCD is thought to trigger plant death and occur in various types of cells and organs for the remobilization of nutrients and secondary metabolites to the developing seeds (Rogers, 2013; Koyama, 2014; Daneva et al., 2016), but no direct evidence has shown that age-induced dPCD occurs in the stem cells of the IM. Our data indicated that the last step of stem cell fate is dPCD, which is associated with vacuolation and BFN1 induction. Future research employing mutants may serve to elucidate in detail the mechanisms of dPCD in the IM.

### ROS Homeostasis May Be a Molecular Switch of Stem Cell Death

It has been reported that ROS levels are associated with dPCD (Daneva et al., 2016; Mhamdi and Van Breusegem, 2018). For instance, ROS accumulation triggers dPCD in the tapetal cells of rice (Yi et al., 2016). Similarly, ROS accumulation, or the H_2_O_2_ burst, was detected in the IM domain at 5 WAB (Figures 5C,D). At 6 WAB, cell death was detected (Figure 5E). These results suggest that the dPCD process in the IM may be triggered by ROS. Based on RNA-seq results and the GO and KEGG enrichment analyses performed in this study, we also isolated a cluster of DEGs, such as peroxiredoxins (*PRX*s) and *catalase 6* (*CAT6*), involved in ROS homeostasis (Figures 5A,B and Supplementary Figure 3), suggesting that these genes might be involved in the dPCD of stem cells in the IM. However, more detailed work, including the analysis of mutants of these factors, is required. Taken together, the findings of previous studies (Mittler et al., 2004; Van Breusegem and Dat, 2006; Zeng et al., 2017) and this study indicate that ROS homeostasis may be a molecular switch of dPCD in stem cells.

### Perspectives for the Research of the IM Longevity in Plants

In this study, we revealed that *CLV3*, PCD, and ROS were involved in the regulation of the longevity of the IM. The IM and inflorescence are useful models for studying the relationship between the longevity of individual organs and that of whole plants. Many questions remain unanswered regarding this subject. For example, what factors promote the dynamic vacuolation of stem cells in the IM? How do plants repress such factors in stem cells until 2 WAB? Furthermore, after the repression of *WUS*, what factors trigger the accumulation of ROS and the expression of *BFN1*? Finally, how do plants coordinate the synchronized death of cells in the IM? Future work may attempt to identify genes and phytohormones controlling plant longevity.

## Data Availability Statement

The datasets generated for this study can be found in DDBJ (https://www.ddbj.nig.ac.jp/dra/index-e.html). ID DRA010789.

## Author Contributions

YW, MS, and TI conceived this study. YW performed all the experiments except RNA-seq library building and sequencing. RNA-seq library building and sequencing were performed by KK, TS, YI, and NY. YW and MS wrote the manuscript. TI and MS revised the original manuscript. All authors read and approved the final version of the manuscript.

## Conflict of Interest

The authors declare that the research was conducted in the absence of any commercial or financial relationships that could be construed as a potential conflict of interest.

## Footnotes

1 https://fiji.sc/

## Supplementary Material

Supplementary Figure 1 Spatiotemporal expression patterns of *WUS* from 4 WAB to 6 WAB. No GUS signals were observed. Scale bars = 50 μm.

Supplementary Figure 2 Volcano plot of DEGs isolated from RNA-seq data sets. The numbers of up- and downregulated DEGs are indicated.

Supplementary Figure 3 Validation of ROS-related DEGs and stem cell marker gene *CLV3* in wild-type plants at 2 WAB and 4 WAB. Blue and pink colors indicate ROS clearance- and ROS production-related DEGs, respectively. Orange color shows the expression of *CLV3*. Error bars denote SD. Two-tailed Student’s *t*-test was performed. ^∗^*p* < 0.05.

Supplementary Figure 4 Histological section shows the observed location of TEM in the *clv3-2* IM domain at 5 WAB. The red box shows the observed area. Scale bar = 50 μm.

Supplementary Figure 5 Magnifying images of side views of *CLV3* expression patterns and FDA/PI staining. **(A)** Magnifying images of side views of *CLV3* expression patterns using *proCLV3:GFP-ER* line (Figure 4C). **(B)** Magnifying images of side views of FDA/PI stained IMs (Figure 5E). White dotted lines indicate SAM shapes. Scale bar = 50 μm in **(A,B)**.

Supplementary Table 1 Primer sequences used in this study.
