# Supplementary material for: Morphological and Physiological Framework Underlying Plant Longevity in Arabidopsis thaliana
Source: Front Plant Sci. 2020 Nov 5;11:600726. doi: 10.3389/fpls.2020.600726 (PMC7674609; doi:10.3389/fpls.2020.600726)
Supplement: Supplementary Figure 1 — Spatiotemporal expression patterns of WUS from 4 WAB to 6 WAB. No GUS signals were observed. Scale bars = 50 μm. [file Data_Sheet_1.pdf]

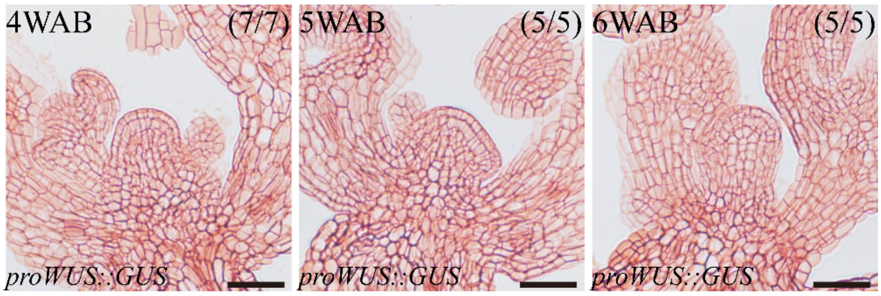

Supplementary Figure 1 | Spatiotemporal expression patterns of *WUS* from 4 WAB to 6 WAB. No GUS signals were observed. Scale bars = 50 μm.

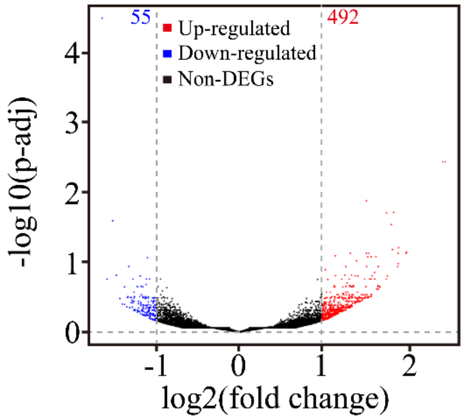

Supplementary Figure 2 | Volcano plot of DEGs isolated from RNA-seq data sets. The numbers of up- and downregulated DEGs are indicated.

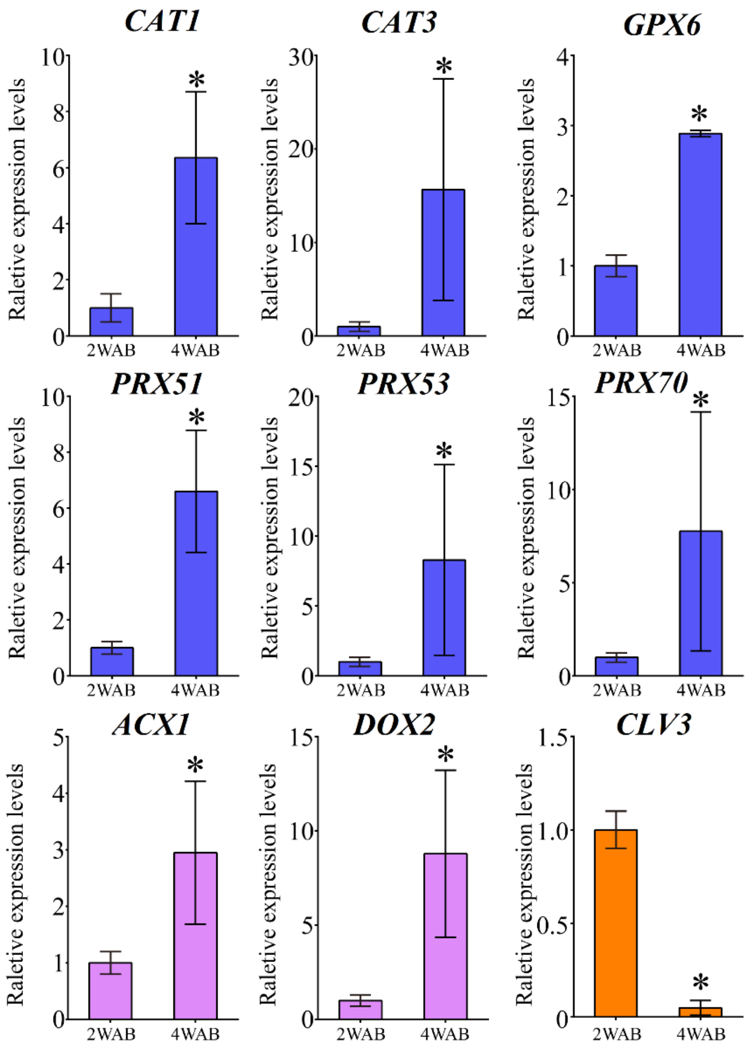

Supplementary Figure 3 | Validation of ROS-related DEGs and stem cell marker gene *CLV3* in wild-type plants at 2 WAB and 4 WAB. Blue and pink colors indicate ROS clearance- and ROS production-related DEGs, respectively. Orange color shows the expression of *CLV3*. Error bars denote S.D. Two-tailed Student's t-test was performed. *: $p < 0.05$.

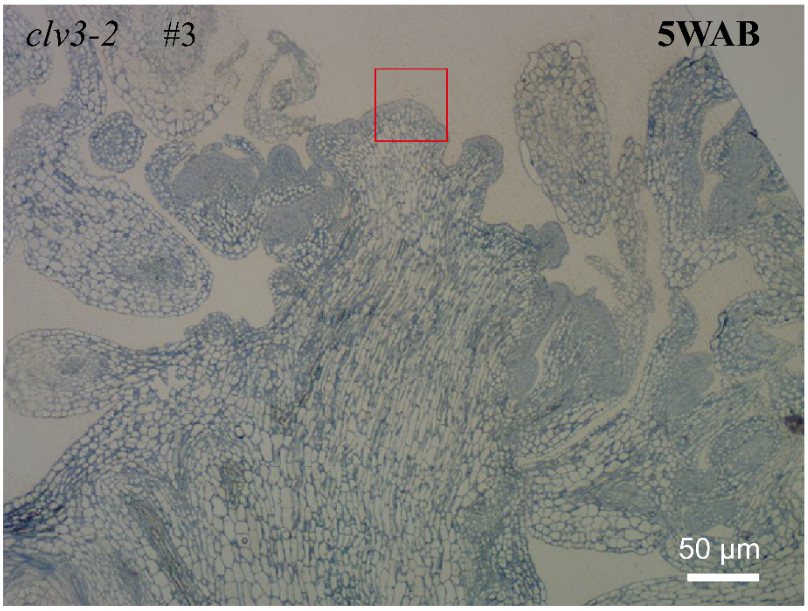

Supplementary Figure 4 | Histological section shows the observed location of TEM in the *clv3-2* IM domain at 5 WAB. The red box shows the observed area. Scale bar = 50 µm.

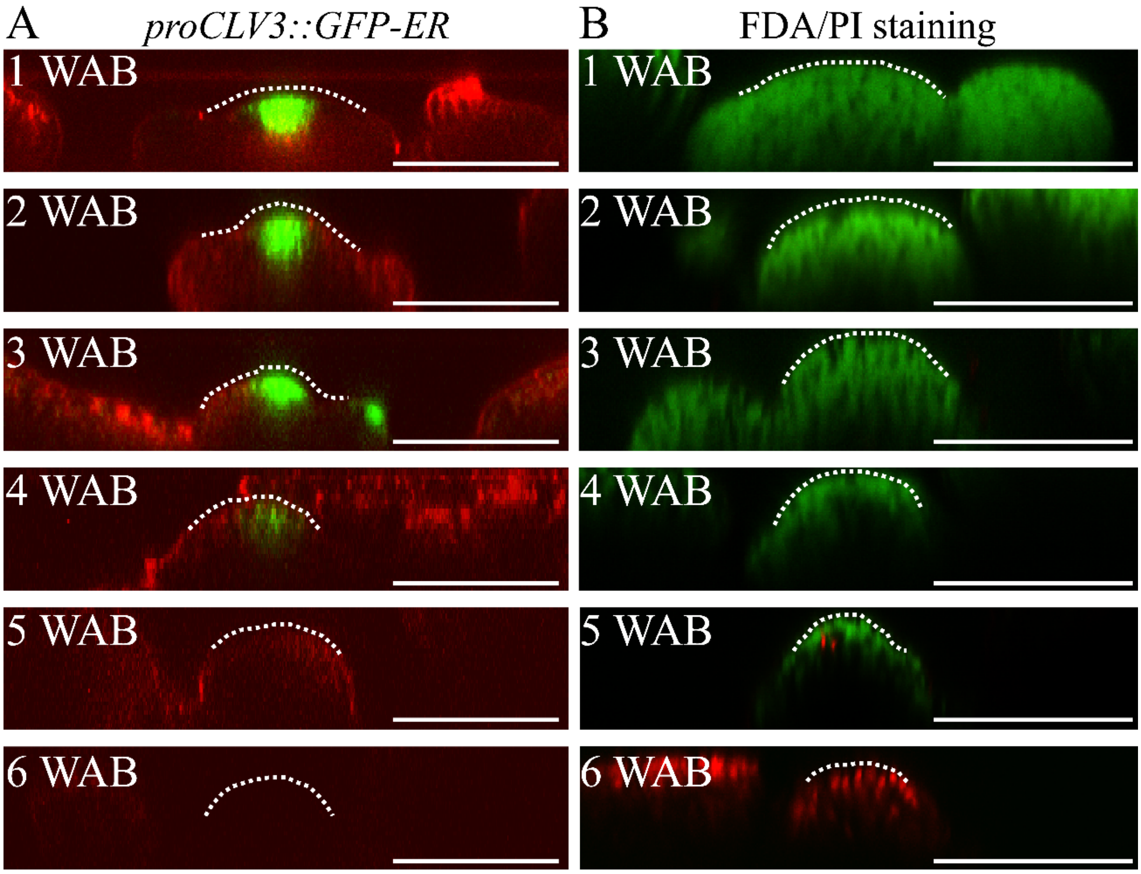

Supplementary Figure 5 | Magnifying images of side views of *CLV3* expression patterns and FDA/PI staining. **(A)** Magnifying images of side views of *CLV3* expression patterns using *proCLV3::GFP-ER* line (Figure 4C). **(B)** Magnifying images of side views of FDA/PI stained IMs (Figure 5E). White dotted lines indicate SAM shapes. Scale bar = 50 μm in **(A)** and **(B)**.

Supplementary Table 1 | Primer sequences used in this study.

Gene name	Forward (5'-3')	Reverse (5'-3')	Annotation
proBFN1	CACCGTTGGAAATTAAGTATTTACCTGC	ATCTTCAAAGTTTGAAACTTATATAATG	Cloning of 2.0 kb promoter
CAT1	GATGATAAGCTACTCCAGACCC	TTGTTGTGGTGAGCACATTTAG	Gene validation by using Real time PCR
CAT3	CTTGTGGTTCCTGGAATCTACT	AGGATCAAACTTTGAGGGGTAG	
GPX6	GGAATCAAGAGCCTGGTACTAA	TTTGTCACCGTTAACATCAACC	
PRX51	GGATTCGACACCGTCATTAAAG	TGAGTTGGTTGAGATCAAAGGT	
PRX53	AAACGCAACATTTTACTCTGGG	CAACAACGTTGAATCCTCTAGC	
PRX70	AGGGACAGATTCTTCAACTACG	AGGTACGACGTATCAAATTGGT	
ACX1	GAGGATATGAAGATCGTCTGGG	TCATTGAGACGAAGCTCGATAA	
DOX2	TATCGACGGAGAAGATAGACCT	TCATCATCTGTCAACTCTTCCC	
CLV3	GTTCAAGGACTTTCCAACCGCAAGATGAT	CCTTCTCTGCTTCTCCATTTGCTCCAACC	
ACTIN2	GAAAAGATCTGGCATCACACTTTATA	ACATACATAGCGGGAGAGTTAAAGGT	Inner reference
